# The Hummingbird Collection of the Natural History and Science Museum of the University of Porto (MHNC-UP), Portugal

**DOI:** 10.3897/BDJ.9.e59913

**Published:** 2021-07-23

**Authors:** Ricardo Jorge Lopes, Pedro Miguel Vieira Faria, Daniela Gomes, Bárbara Freitas, Judit Málinger

**Affiliations:** 1 MHNC-UP, Natural History and Science Museum of the University of Porto, Porto, Portugal MHNC-UP, Natural History and Science Museum of the University of Porto Porto Portugal; 2 CIBIO, Centro de Investigação em Biodiversidade e Recursos Genéticos, InBIO Laboratório Associado, Campus Agrário de Vairão, Vairão, Portugal CIBIO, Centro de Investigação em Biodiversidade e Recursos Genéticos, InBIO Laboratório Associado, Campus Agrário de Vairão Vairão Portugal; 3 FCUP, Faculdade de Ciências da Universidade do Porto, Porto, Portugal FCUP, Faculdade de Ciências da Universidade do Porto Porto Portugal; 4 University of Pannonia, Veszprem, Hungary University of Pannonia Veszprem Hungary

**Keywords:** Hummingbirds, Trochilidae, museum, biodiversity information databases, specimen

## Abstract

**Background:**

The Hummingbird (Family Trochilidae) Collection of the Natural History and Science Museum of the University of Porto (MHNC-UP) is one of the oldest collections of this family harboured in European museums. Almost 2,000 specimens, that encompass most of the taxonomic diversity of this family, were collected in the late 19^th^ Century. The collection is relevant due its antiquity and because all specimens were bought from the same provider, mainly as mounted specimens, for a Portuguese private collection of Neotropical fauna. In the early 20^th^ Century, it was donated to the Museum that is now the MHNC-UP.

**New information:**

The information about the majority of these specimens is now available for consultation on the GBIF platform after curation of all specimens and digital cleaning of the associated metadata. In the process, hundreds of non-catalogued specimens were found and taxonomic and spatial information was updated for many of the specimens.

## Introduction

The largest zoological collections in contemporary Portugal are currently curated by three main museums, in Lisbon, Coimbra and Porto (Lourenço and Dias 2017). In Porto, the new MHNC-UP (Museum of Natural History and Science of the University of Porto) has its origins in the establishment of the Cabinet of Natural History of the Polytechnic Academy of Porto (1837), although it was already its successor, the Faculty of Sciences of the University of Porto, that opened the collections for the public in 1916 (Delicado 2010, Ceríaco 2014). The MHNC-UP was formally established at the end of 2015 and is the result of the fusion of several museums and collections of the University of Porto, including the “Instituto de Zoologia Augusto Nobre”, along with the private collection of “Museu Braga Júnior”. This is now the institution responsible for the preservation and study of these specimens, which also includes a vast number of assets in various fields of Science and Natural History, ranging from Geology, Zoology and Botany collections to ethnological, paleontological and archaeological artefacts and scientific instruments. The bird collection holds a major percentage of the vertebrate specimens in the Museum (more than 80% of all specimens), comprising one of the largest collections of its kind in Portugal, with more than 7,000 specimens.

In the scope of the fundamental mission of Natural History Museums (Bakker et al. 2020, Cook et al. 2014, Lister and Climate Change Research Group 2011, Shaffer et al. 1998) to preserve current and historic data from biological, geological, paleontological and other natural history sources, our primary goal is the preservation of these specimens and their metadata, due to their value for various fields of science (Genetics, Systematics, Taxonomy, Evolution, Phylogeography, amongst others). This data paper is the result of the ongoing policy of curating the most important information, including taxonomic, geographical and temporal data, which were, in many instances, outdated or misplaced. This initiative included the reconciliation of the information in the database for each specimen, correcting taxonomic information, if necessary and updating the specimen identification.

**Figure 1.**
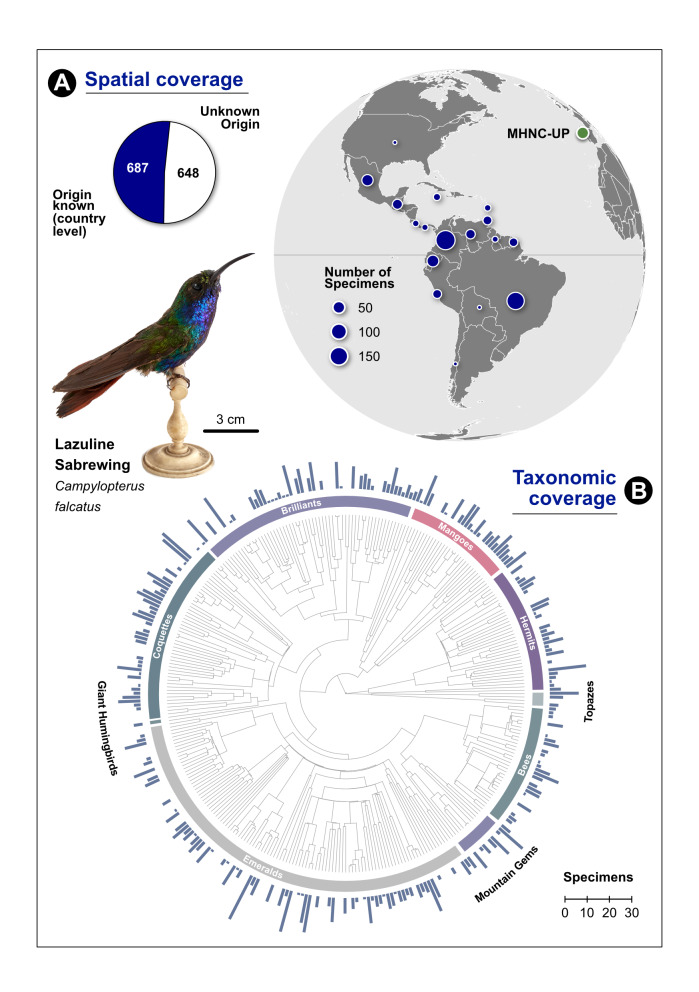
Spatial and taxonomic coverage of the Hummingbird Collection. An example is also shown of a mounted specimen with pedestal, illustrating the conditions of the majority of the collection. A) The number of specimens with spatial data and with known origin to the country level; B) Phylogenetic tree of the family Trochilidae with the number of specimens in the MHNC-UP collection of each species as bars. The major groups (see Introduction) are outlined by coloured clade arches. We used the hummingbird species dataset from BirdTree.org to build a maximum clade credibility tree using TreeAnnotator (Bouckaert et al. 2019). Final tree and bars were drawn using “phytools” and “ggtree” packages (Yu et al. 2018, Revell 2012) in the R statistical environment (R Core Team 2019).

The Hummingbirds (Family Trochilidae) are the most representative group in the MHNC-UP Bird Collection, as they account for more than 20% of all birds and hold a considerable number of species, representative of almost all genera of this family (Fig. 1). Almost two thousand specimens were collected in the late 19^th^ Century and they are one of the oldest collections of this family harboured in European museums. It is highly relevant that they were mainly bought as mounted specimens, from the same provider, to be the core of a private collection of Neotropical birds. Ultimately, it was donated in the early 20^th^ Century to the Museum that is now the MHNC-UP.

Hummingbirds are a remarkably distinct group of birds, classified as members of the order Caprimulgiformes, along with nightjars, swifts and tree swifts, amongst others (Schuchmann 1999, McGuire et al. 2014). They occur throughout the Americas, most species concentrating in South and Central America in a variety of habitats, with a preference for tropical and sub-tropical conditions. Recent phylogenetic analyses of all 102 genera and 324 species of currently recognised hummingbirds corroborate nine strongly-supported clades: Topazes, Hermits, Mangoes, Brilliants, Coquettes, Giant Hummingbirds, Mountain Gems, Bees and Emeralds (McGuire et al. 2009, McGuire et al. 2014).

Beyond their intrinsic taxonomic value, hummingbird specimens possess several characteristics that makes them valuable for exploring evolutionary and ecological concepts (Altshuler and Dudley 2002, Suarez and Gass 2002). First, they show great taxonomic diversity, with the second greatest number of species of any bird family (after the tyrant flycatchers). Second, they show great morphologial diversity, with large differences in body, bill size and shape and also large variations on colouration. These characteristics allow the possibility of robust comparative studies. For example, this large diversity is attributed by several authors to co-evolution with the plants they depend upon for foraging (Abrahamczyk et al. 2015, Dalsgaard et al. 2009, McGuire et al. 2014, Stiles 1978). In addition, this large number of species allows comparative analysis of the evolution of their plumage colouration (Ornelas et al. 2016, Eliason et al. 2020).

Our ultimate goal includes the digitalisation of all hummingbird specimens, including photographic records and display of specimens, both in physical form through the MHNC-UP exhibitions and in digital form, through publishing of corrected and complete data in the global database GBIF, making it available for consultation by researchers all over the world (Edwards 2004).

## General description

### Purpose

The Hummingbird (Trochilidae family) specimens comprise the group with the highest number of species and specimens in the MHNC-UP Bird Collection, with almost two thousand specimens from a considerable number of species (242), representative of almost all genera of this family (Fig. 1). Its value is quite high, given their age and rarity of some of these specimens and the ethical and logistics concerns of harvesting new birds. The majority of the specimens (1335) are mounted on white painted wood pedestals (Fig. 1) and were prioritised in the first phase of curation of this collection. However, skins (~150), nests with eggs (~50) and a diorama with more than one hundred specimens perched in a small tree are also present in the Collection and will be added in the second phase.

### Additional information

The Hummingbird Collection holds 35 specimens from 15 species (Table 1), listed in threatened categories by IUCN (International Union for Conservation of Nature). Of these, five species are Critically Endangered, seven are Endangered and three are Vulnerable. The presence of the Critically Endangered (possibly extinct) Turquoise-throated Puffleg is significant (*Eriocnemis godini* (Bourcier, 1851)) (Schuchmann et al. 2001, Schuchmann 1999). It is only known by six specimens which were collected in the 19^th^ century, all recent surveys failed to find individuals and IUCN believes it may be extinct (BirdLife International 2016). Also of high relevance are the two specimens of the Critically-Endangered Blue Bearded Helmetcrest (*Oxypogon cyanolaemus*, Salvin & Godman, 1880), a species with a population size lower than 250 individuals, that inhabits high altitude habitats in northern Colombia, last recorded around 70 years ago (Collar and Salaman 2013, BirdLife International 2018, Rojas and Vasquez 2015).

## Sampling methods

### Study extent

The Collection covers ten countries (United States, Mexico, Panama, Colombia, Venezuela, Trinidad and Tobago, French Guiana, Brazil, Ecuador and Peru) of the American Continent (Fig. 1).

### Sampling description

This Collection was mainly collected through a well-regarded French Naturalist supplier (Deyrolle) in terms of taxidermy and entomology, since its inception in 1831 (Fox 2012). Unfortunately, there is no information available regarding the original collectors, either at the MHNC-UP or from Deyrolle documentation. José Teixeira da Silva Braga Júnior purchased a vast collection of these tropical hummingbirds in the period from 1875 to 1904 for his own private museum in the “Palacete Braguinha”, that is now FBAUP (Faculdade de Belas Artes da Universidade do Porto). However, in 1928, the legal heirs donated the Collection to the Museum that would come nowadays to be known as the MHNC-UP.

### Quality control

The collection of hummingbirds of the MHNC-UP was first catalogued in digital format after 2005 using a proprietary relational database software (Index Rerum, FCo. São João da Madeira). In 2016-18, we transcribed the catalogued information, revised or updated data and, finally, imported the dataset into a new relational database.

The first priority of the curatorial process was to correct taxonomic and geographic data for each specimen, using validation lists for taxa and locations. For the taxonomic information, we used the latest HBW and BirdLife Taxonomic Checklist, version v.4 (del Hoyo and Collar 2014), due to is wide acceptance and because it matches the species evaluated in the IUCN Red List of Threatened Species. This checklist includes information on the scientific and common names used by BirdLife, the Authority (for the original description of the taxon), BirdLife’s taxonomic treatment (recognised, not recognised or under review), the latest IUCN Red List category (e.g. Extinct, Vulnerable, Least Concern), the sources that recognise the taxon, the scientific and common names used by these sources, where relevant, a taxonomic note and a record ID number unique to the taxonomic entity. When the species name did not match any name on the list, a process of data reconciliation was initiated, that relied on several bibliographical sources (Fogden et al. 2014, de Schauensee and Phelps 1978, Williamson 2002, Schuchmann 1999) and internet sources of digital information (e.g. Avibase; Birds of the World; Wiki Aves) to match the old and synonym species names already in the database with the contemporary species name. For locations, following the Named Area Standards recommended by GBIF, we followed ISO3166, a standard published by the International Organisation for Standardisation (ISO) that defines codes for the names of countries, dependent territories, special areas of geographical interest and their principal subdivisions (e.g. provinces or states). Geographical information was validated using the location list to the Country level.

### Step description

The process of curation started with the establishment of a match between the digital data and the specimen. Then, using archival paper tags, each specimen’s main information (Museum unique catalogue number, Species Identification and gender and spatial data, when available) was transcribed and the tag attached to one leg. Then, a digital photograph was taken, to provide a long-term digital voucher of the specimen and the tag. Final files were saved as a JPEG file and named after the Catalogue number, unique to each specimen, along with EXIF data (Scientific name and Catalogue number), so that a direct link to the database would be easy to accomplish and verify.

## Geographic coverage

### Description

This Collection holds multiple specimens from almost the entire range of hummingbirds in the American Continent, including multiple islands (e.g. Trinidad and Tobago). However, the coverage is much lower than the actual distribution of the specimens, since almost half of the collection (648 specimens - 48%) is not referenced to country level and precise geographic location is only available for a very small subset - 75 specimens (Fig. 1). A further issue is the common practice in the 19^th^ Century of providing the name of major cities as the location, especially when local collectors were involved and it is now difficult to discern specimens that were truly recorded near a capital and those that were not. Although most species have a geographical distribution larger than one single country, for those species endemic at the country level, it was possible to increase the precision to the country level.

## Taxonomic coverage

### Description

Concerning the taxonomic representation, MHNC-UP’s Hummingbird Collection covers all the evolutionary ranges of this family (Fig. 1), including most of the genus, making it valuable for studies pertaining to taxonomy, systematics or phylogeny of the Hummingbird family. In addition, for more than 15% of the species, the number of specimens is higher than 10, with representatives of both genders and life stages (adult and juveniles) (Fig. 1).

### Taxa included

**Table:** 

Rank	Scientific Name	
kingdom	Animalia	
phylum	Chordata	
class	Aves	
order	Caprimulgiformes	
family	Trochilidae	Hummingbirds

## Temporal coverage

**Living time period:** 1850-1900.

### Notes

Unfortunately, there are no records of sampling or preparation dates. However, the specimens were bought most probably in batches, by the same client, to Deyrolle, an institution founded in 1831 in Paris, that soon established a reputation as a taxidermist and provider of specimens for many museums and private collections (de Broglie and Polle 2017). An enquiry about the existence of records on Deyrolle logbooks proved to be unfruitful, since most of the records were lost in a major fire in 2008. The owner, José Teixeira da Silva Braga Júnior established his collection at Porto only after the purchase of the former Forbes Palace by his father in 1875 and he died in1904. Considering these facts, we estimate that the temporal range of the records lies between 1850 and 1900.

## Collection data

### Collection name

Hummingbirds

### Parent collection identifier

MHNCUP/AVE

## Usage licence

### Usage licence

Creative Commons Public Domain Waiver (CC-Zero)

## Data resources

### Data package title

The Hummingbird Collection of the Natural History and Science Museum of the University of Porto (MHNC-UP)

### Resource link

https://www.gbif.org/dataset/17f6f3d0-b5ef-434b-95a8-cc32d8e5236f

### Alternative identifiers

https://doi.org/10.15468/6x9lng

### Number of data sets

1

### Data set 1.

#### Data set name

The Hummingbird Collection of the Natural History and Science Museum of the University of Porto (MHNC-UP)

#### Number of columns

41

#### Description

The Hummingbird (Family Trochilidae) Collection of the Natural History and Science Museum of the University of Porto (MHNC-UP) is one of the oldest collections of this family harboured in European museums. Almost two thousand specimens, that encompass most of the taxonomic range of this family, were collected in the late 19^th^ Century. They were bought from the same provider, mainly as mounted specimens, for a Portuguese private collection that was donated in the 20^th^ Century to the Museum that is now MHNC-UP. The information about these specimens is now available for consultation on the GBIF platform (Lopes et al. 2019).

**Data set 1.** 

Column label	Column description
type	The nature or genre of the resource.
modified	The most recent date-time on which the resource was changed.
language	A language of the resource.
license	A legal document giving official permission to do something with the resource.
rightsHolder	A person or organisation owning or managing rights over the resource.
accessRights	Information about who can access the resource or an indication of its security status.
institutionID	An identifier for the institution having custody of the object(s) or information referred to in the record.
institutionCode	The name (or acronym) in use by the institution having custody of the object(s) or information referred to in the record.
collectionID	An identifier for the collection or dataset from which the record was derived.
collectionCode	The name, acronym, coden or initialism identifying the collection or dataset from which the record was derived.
datasetName	The name identifying the dataset from which the record was derived.
basisofRecord	The specific nature of the data record.
occurrenceID	An identifier for the Occurrence (as opposed to a particular digital record of the occurrence).
CatalogNumber	An identifier (preferably unique) for the record within the dataset or collection.
recordedBy	A list of names of people, groups or organisations responsible for recording the original Occurrence.
individualCount	The number of individuals represented present at the time of the Occurrence.
preparations	A list of preparations and preservation methods for a specimen
sex	The sex of the biological individual(s) represented in the Occurrence.
lifeStage	The age class or life stage of the biological individual(s) at the time the Occurrence was recorded.
preparations	A list of preparations and preservation methods for a specimen.
disposition	The current state of a specimen with respect to the collection identified in collectionCode or collectionID.
otherCatalogNumbers	A list of previous or alternate fully qualified catalogue numbers or other human-used identifiers for the same Occurrence, whether in the current or any other dataset or collection.
previousIdentifications	A list (concatenated and separated) of previous assignments of names to the Organism.
eventDate	The date-time or interval during which an Event occurred.
continent	The name of the continent in which the Location occurs.
island	The name of the island on or near which the Location occurs.
country	The name of the country or major administrative unit in which the Location occurs.
countryCode	The standard code for the country in which the Location occurs.
locality	The specific description of the place.
verbatimLocality	The original textual description of the place.
scientificName	The full scientific name, with authorship and date information if known.
kingdom	The full scientific name of the kingdom in which the taxon is classified.
phylum	The full scientific name of the phylum in which the taxon is classified.
class	The full scientific name of the class in which the taxon is classified.
order	The full scientific name of the order in which the taxon is classified.
family	The full scientific name of the family in which the taxon is classified.
genus	The full scientific name of the genus in which the taxon is classified.
specificEpithet	The name of the species epithet of the scientificName.
taxonRank	The taxonomic rank of the most specific name in the scientificName.
taxonomicStatus	The status of the use of the scientificName as a label for a taxon.
taxonRemarks	Comments or notes about the taxon or name.

## Figures and Tables

**Table 1.** Species in the Hummingbird Collection considered by IUCN (International Union for Conservation of Nature) to be Vulnerable, Endangered or Critically Endangered and number of specimens (n).

**Scientific Name**	**Common Name**	**Conservation Status**	**n**
*Eriocnemis godini*	Turquoise-throated Puffleg	Critically Endangered (Probably extinct)	3
*Eriocnemis nigrivestis*	Black-breasted Puffleg	Critically Endangered	2
*Eulidia yarrellii*	Chilean Woodstar	Critically Endangered	1
*Oxypogon cyanolaemus*	Blue-bearded Helmetcrest	Critically Endangered	2
*Sephanoides fernandensis*	Juan Fernandez Firecrown	Critically Endangered	3
*Aglaiocercus berlepschi*	Venezuelan Sylph	Endangered	1
*Amazilia castaneiventris*	Chestnut-bellied Hummingbird	Endangered	1
*Campylopterus phainopeplus*	Santa Marta Sabrewing	Endangered	1
*Hylonympha macrocerca*	Scissor-tailed Hummingbird	Endangered	4
*Loddigesia mirabilis*	Marvellous spatuletail	Endangered	1
*Metallura baroni*	Violet-throated Metaltail	Endangered	1
*Thalurania watertonii*	Long-tailed Woodnymph	Endangered	4
*Chaetocercus bombus*	Little woodstar	Vulnerable	1
*Coeligena prunellei*	Black inca	Vulnerable	5
*Lophornis gouldii*	Dot-eared coquette	Vulnerable	5
